# Anti-cancer activity of guggulsterone by modulating apoptotic markers: a systematic review and meta-analysis

**DOI:** 10.3389/fphar.2023.1155163

**Published:** 2023-05-02

**Authors:** Meenakshi Gupta, Deepti Singh, Shruti Rastogi, Hifzur R. Siddique, Noura Al-Dayan, Ajaz Ahmad, Mohammad Sikander, Maryam Sarwat

**Affiliations:** ^1^ Amity Institute of Pharmacy, Amity University, Noida, Uttar Pradesh, India; ^2^ Molecular Cancer Genetics & Translational Research Lab, Section of Genetics, Department of Zoology, Aligarh Muslim University, Aligarh, India; ^3^ Indian Pharmacopoeia Commission, Ministry of Health & Family Welfare, Government of India, Ghaziabad, Uttar Pradesh, India; ^4^ Medical Laboratory Department, Applied Medical Science, Prince Sattam Bin Abdul Aziz University, Al-Kharj, Saudi Arabia; ^5^ Department of Clinical Pharmacy, College of Pharmacy King Saud University, Riyadh, Saudi Arabia; ^6^ Department of Immunology and Microbiology, Biomedical Research, The University of Texas, McAllen, TX, United States

**Keywords:** guggulsterone, meta-analysis, systematic literature review, apoptosis, anti-cancer

## Abstract

**Background:** Guggulsterone (pregna-4,17-diene-3,16-dione; C_21_H_28_O_2_) is an effective phytosterol isolated from the gum resin of the tree *Commiphora wightii* (Family Burseraceae) and is responsible for many of the properties of guggul. This plant is widely used as traditional medicine in Ayurveda and Unani system of medicine. It exhibits several pharmacological activities, such as anti-inflammatory, analgesic, antibacterial, anti-septic and anticancer. In this article, the activities of Guggulsterone against cancerous cells were determined and summarized.

**Methods:** Using 7 databases (PubMed, PMC, Google Scholar, Science Direct, Scopus, Cochrane and Ctri.gov), the literature search was conducted since conception until June 2021. Extensive literature search yielded 55,280 studies from all the databases. A total of 40 articles were included in the systematic review and of them, 23 articles were included in the meta‐analysis.The cancerous cell lines used in the studies were for pancreatic cancer, hepatocellular carcinoma, head and neck squamous cell carcinoma, cholangiocarcinoma, oesophageal adenocarcinoma, prostrate cancer, colon cancer, breast cancer, gut derived adenocarcinoma, gastric cancer, colorectal cancer, bladder cancer, glioblastoma, histiocytic leukemia, acute myeloid leukemia and non-small cell lung cancer. The reliability of the selected studies was assessed using ToxRTool.

**Results:** Based on this review, guggulsterone significantly affected pancreatic cancer (MiaPaCa-2, Panc-1, PC-Sw, CD18/HPAF, Capan1, PC-3), hepatocellular carcinoma (Hep3B, HepG2, PLC/PRF/5R), head and neck squamous cell carcinoma (SCC4, UM-22b, 1483), cholangiocarcinoma (HuCC-T1, RBE, Sk-ChA-1, Mz-ChA-1) and oesophageal adenocarcinoma (CP-18821, OE19), prostrate cancer (PC-3), colon cancer (HT-29), breast cancer (MCF7/DOX), gut derived adenocarcinoma (Bic-1), gastric cancer (SGC-7901), colorectal cancer (HCT116), bladder cancer (T24, TSGH8301), glioblastoma (A172, U87MG, T98G), histiocytic leukemia (U937), acute myeloid leukemia (HL60, U937) and non-small cell lung cancer (A549, H1975) by inducing apoptotic pathways, inhibiting cell proliferation, and regulating the expression of genes involved in apoptosis. Guggulsterone is known to have therapeutic and preventive effects on various categories of cancers. It can inhibit the progression of tumors and can even reduce their size by inducing apoptosis, exerting anti-angiogenic effects, and modulating various signaling cascades. *In vitro* studies reveal that Guggulsterone inhibits and suppresses the proliferation of an extensive range of cancer cells by decreasing intrinsic mitochondrial apoptosis, regulating NF-kB/STAT3/β-Catenin/PI3K/Akt/CHOP pathway, modulating the expression of associated genes/proteins, and inhibiting angiogenesis. Furthermore, Guggulsterone reduces the production of inflammatory markers, such as CDX2 and COX-2. The other mechanism of the Guggulsterone activity is the reversal of P-glycoprotein-mediated multidrug resistance. Twenty three studies were selected for meta-analysis following the PRISMA statements. Fixed effect model was used for reporting the odds ratio. The primary endpoint was percentage apoptosis. 11 of 23 studies reported the apoptotic effect at t = 24 h and pooled odds ratio was 3.984 (CI 3.263 to 4.865, *p* < 0.001). 12 studies used Guggulsterone for t > 24 h and the odds ratio was 11.171 (CI 9.148 to 13.643, 95% CI, *p* < 0.001). The sub-group analysis based on cancer type, Guggulsterone dose, and treatment effects. Significant alterations in the level of apoptotic markers were reported by Guggulsterone treatment.

**Conclusion:** This study suggested that Guggulsterone has apoptotic effects against various cancer types. Further investigation of its pharmacological activity and mechanism of action should be conducted. *In vivo* experiments and clinical trials are required to confirm the anticancer activity.

## 1 Introduction

Cancer is a multi-stage disease characterized by replicative immortality, an aberration in the signaling circuitry, and evasion of the immune system. Risk factors include sex, race, genetics, epigenetics, lifestyle, nutrition, obesity, smoking, and alcohol consumption. Currently, available treatment strategies include radiotherapy, surgical resection, stem cell transplant, hormone therapy, immunotherapy, targeted therapy, and chemotherapy. Management with the current regimen is associated with severe side effects like hair loss, bleeding, bruising, nausea, vomiting, and fatigue and leaves the relevant population without effective therapy. Major limitations of existing strategies are cancer recurrence, poor selectivity towards tumor tissues, and multi-drug resistance against chemotherapeutic agents. These limitations restrain the use of chemotherapeutic drugs and impair the patient’s quality of life (Abdulridha et al., 2020). Provided that the cases of cancer are on the rise and the treatment is expensive and less effective, it is decisive to explore economically viable and potent methods for the patients. The need for the identification of novel and promising anticancer agents having better efficacy and lesser side effects continues.

**FIGURE 1:**
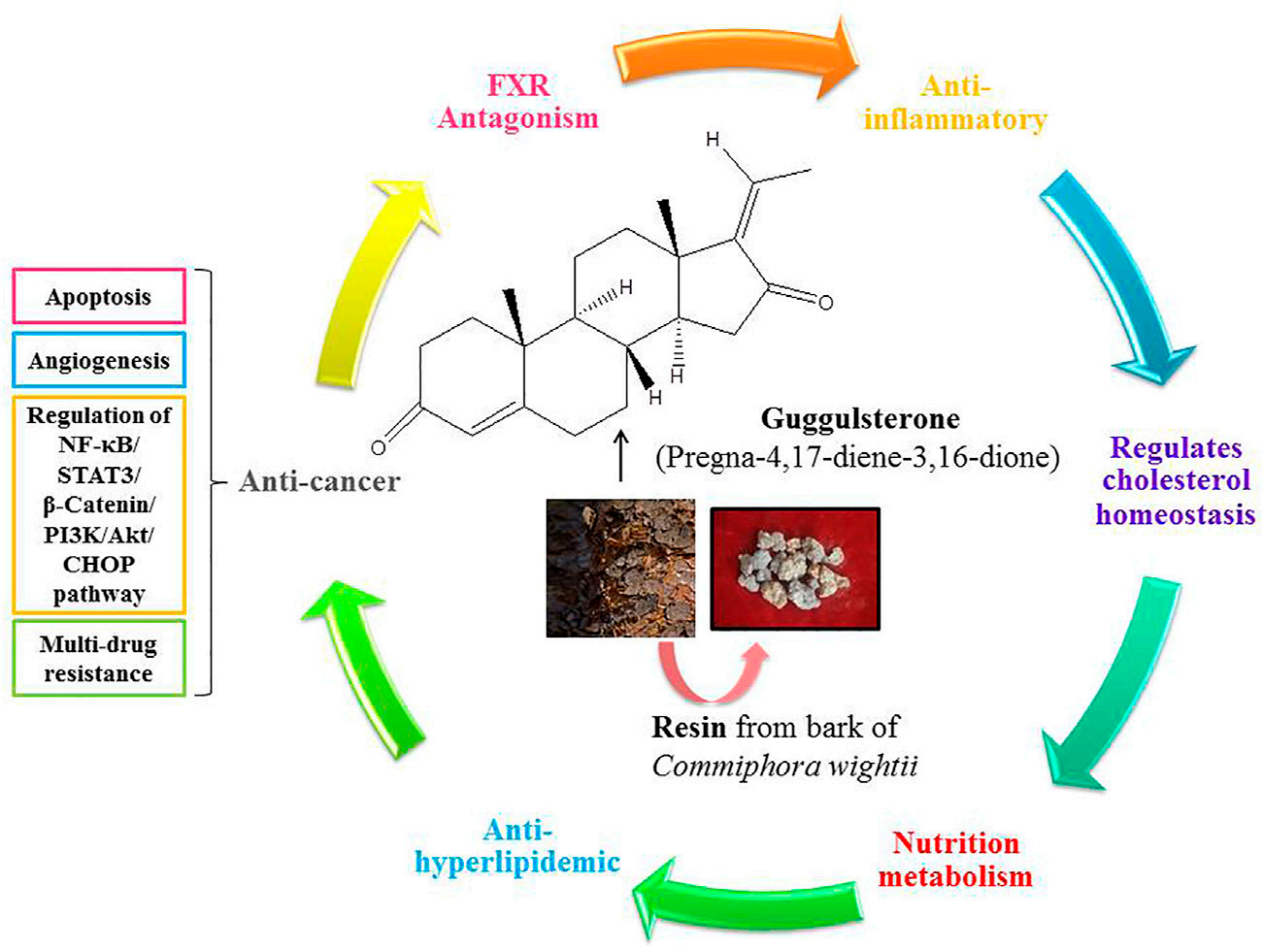
Schematic representation of the pleiotropic effects of Guggulsterone.

In recent times, herbal medicines have unraveled pleiotropic effects in the treatment of various diseases and have gained enormous attention. Extraction of these phytoconstituents and gaining detailed information about the performance of these compounds for the management of different types of cancer and various other diseases has become the center of attraction among researchers. Amongst the wide range of medicinal herbs, Guggulsterone (pregna-4,17-diene-3,16-dione; C_21_H_28_O_2_) is an effective phytosterol isolated from the gum resin of the tree *Commiphora wightii* and is responsible for many of the properties of guggul (Figure 1). Guggulsterone has been proven to be an antagonist ligand for the farnesoid X receptor (FXR) and to suppress the expression of FXR agonist-induced genes (Bhat et al., 2017).

**FIGURE 2:**
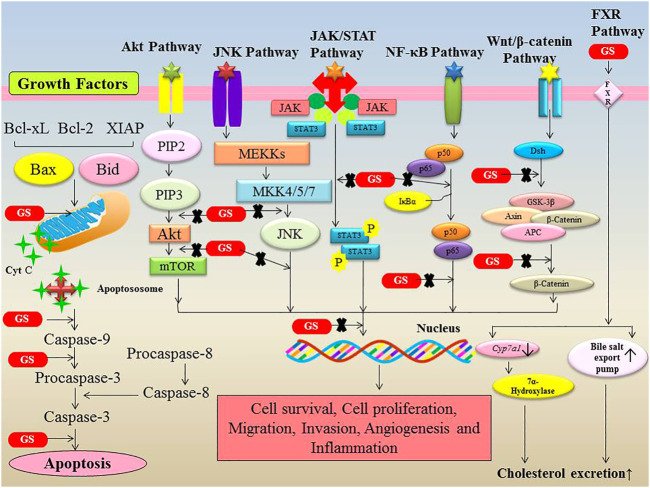
Modulation of various signalling pathways involved in cancer by Guggulsterone. Guggulsterone modulates the expression of numerous proteins involved in cell survival, cell proliferation, migration, invasion, angiogenesis and inflammation.

FXR is a bile acid nuclear receptor that regulates the expression of key genes involved in bile acid and cholesterol homeostasis. FXR inhibits the expression of cholesterol 7-hydroxylase (Cyp7a1), sterol 12-hydroxylase, the Na/taurocholate co-transporting polypeptide, and apolipoprotein A-I, while activating the expression of intestinal bile acid-binding protein (I-BABP), bile salt export pump (BSEP), apolipoprotein C-II, phospholipid transfer protein, and dehydroepiandrosterone sulfotransferase. Guggulsterone also have a significant role in nutritional metabolism by inhibiting cholesterol production in the liver via FXR and bile-acid receptor antagonism (Chiang et al., 1990; Owsley and Chiang, 2003). Guggulsterone effectively lowers low density lipoprotein cholesterol and triglyceride levels in the blood while increasing high density lipoprotein cholesterol levels. The cholesterol homeostasis regulating, anti-hyperlipidemic and nutritional metabolism maintaining properties of guggulsterone are attributable to the inhibition of FXR (Kunnumakkara et al., 2018). Increased FXR expression has been reported to positively influence cancer cell proliferation and tumour development in non-small cell lung cancer (NSCLC), which may include the activation of multiple oncogenes such as cyclin D1. FXR, on the other hand, works as a tumour suppressor in intestinal tumours, and its lack of expression leads to accelerated tumour development. It implies that FXR has a dual role as a proto-oncogene or tumour suppressor gene depending on its tissue function. The FXR protein is also implicated in the regulation of other molecules, including TNF-α, p21, Bcl-2, nuclear factor kappa-B (NF-κB), and other pro-inflammatory cytokines. Guggulsterone is known to have therapeutic and preventive effects on various categories of cancers. It can inhibit the progression of tumors and can even reduce their size by inducing apoptosis, exerting anti-angiogenic effects, and modulating various signaling cascades (Bhat et al., 2017; Kunnumakkara et al., 2018). *In vitro* studies reveal that Guggulsterone inhibits and suppresses the proliferation of an extensive range of cancer cells by decreasing intrinsic mitochondrial apoptosis, regulating NF-κB/STAT3/β-Catenin/PI3K/Akt/CHOP pathway, modulating the expression of associated genes/proteins, and inhibiting angiogenesis. Furthermore, Guggulsterone reduces the production of inflammatory markers, such as CDX2 and COX-2 (Figure 2). The other mechanism of the Guggulsterone activity is the reversal of P-glycoprotein-mediated multidrug resistance (Bhat et al., 2017; Kunnumakkara et al., 2018).

Another nuclear receptor, liver-X-receptor-α (LXR-α), play a role in cholesterol homeostasis, and LXR-α activation has an anti-inflammatory impact by suppressing NF-κB-mediated signalling. Although FXRs respond to bile acids and LXRs to oxysterol molecules within the cellular nucleus, their coordinated ligand-specific activities stimulate transcription and change the expression patterns of numerous genes. Genes, in particular, are in charge of cholesterol, lipid, bile acid, and glucose metabolism, as well as general liver function (Shiragannavar et al., 2023).

Nowadays, the anti-cancer effect of Guggulsterone is a matter of interest in experimental research. However, there is very less information that suggests the use of Guggulsterone for pre-clinical and clinical studies. Systematic reviews and meta-analysis can therefore help clarify whether this molecule could be beneficial in the management of various cancer types and decide if Guggulsterone could be explored further on animal and clinical grounds. To date, no study has systematically synthesized nor critically appraised the original research studies that have explored the impact of Guggulsterone in cancer cell growth and metastasis. Therefore, the present study was constructed to investigate the therapeutic effect of Guggulsterone in the management of various cancers in studies done on various cancer cell lines.

## 2 Methods

### 2.1 Data sources and searches

The review followed the recommendations in the Preferred Reporting Items for Systematic Reviews and Meta-analyses (PRISMA) Statement. The literature search was conducted until June 2021. The following databases were searched: PubMed, PMC, Google Scholar, Science Direct, Scopus, Cochrane and Ctri.gov. For the title or abstract, the following keywords were used: sets “Guggulsterone”, “*Commiphora mukul*”, and “*Commiphora wightii*”. The following strings were also used in combination to ensure maximum capture of the literature “HCC”, “Hepatocellular carcinoma”, “Liver cancer”, “Cancer”, “Cirrhosis”, and “Fibrosis”. The bibliographic reference list of eligible studies was also checked. The final search result from each database was limited to the studies published in the English language.

### 2.2 Inclusion and exclusion criteria

Inclusion and exclusion criteria were identified according to the Population, Intervention, Control, and Outcomes (PICO) principle. We included the *in vitro* studies where Guggulsterone was tested against a control arm (vehicle). There was no limitation on the type of cancer, dosage of Guggulsterone, or duration of treatment. Based on the title, abstracts, and, full manuscripts, the data extraction, and quality assessment were done by the independent reviewers (MG and DS). The studies were based on the following criteria: i) Guggulsterone used as a major intervention, ii) as an anti-cancer therapeutic, and iii) focusing on the effect on apoptosis and apoptotic pathways. The following studies were excluded: i) papers focused on studies other than cancer; ii*) in silico* studies; iii) human studies; iv) *in vivo* studies v) review articles, vi) studies having inadequate information; vii) studies in which Guggulsterone was not used as a major intervention and viii) studies not published in English.

### 2.3 Intervention

All the experimental groups treated with different doses of Guggulsterone and demonstrating an increase in the percentage of apoptotic cells as compared to control were considered. In addition, the treated group also revealed alteration in the expression of apoptotic markers such as Bcl-2, Bcl-xL, Mcl-1, Bax, Bad, Bak, Bid, Fas, cIap-1/2, Survivin, and Caspase-3/8/9 were considered. If different doses of Guggulsterone were used in the study, the highest dose was chosen for performing a meta-analysis.

### 2.4 Data extraction and quality assessment

Data from the included studies were extracted independently by two authors (MG and DS) and cross-checked to avoid any discrepancies with a third author (SR). Other authors verified the same and gave the final confirmation. Studies that assess the therapeutic effects of Guggulsterone against various cancer types were included in the review. The following data were extracted: author’s name, year of publication, cancer type, study design, the cell line used, type of intervention, dosage, duration, genes upregulated or downregulated, and the pathway of action. To ensure consistency, all the data was summarized in a structured table.

Two independent reviewers (MG and DS) performed the quality assessment of studies using the *in-vitro* ToxRTool. This tool provides a detailed and transparent evaluation of the ecotoxicity data. Individual quality items were examined using the 5 criteria groups: 1) test substance identification, (2) test system characterization, (3) study design description, (4) study results documentation, and (5) plausibility of study design and data. A score of 0 (‘no-not met’) or 1 (‘yes-met’) was assigned to the sub-criteria by two reviewers (MG and DS) while evaluating the study. A combined score was calculated for each rater and each study, by summing the scores of the above-mentioned criteria of each selected study. The following approach was used to grade the quality of each study as category 1 (Score15-18; reliable without restrictions), category 2 (Score11-14; reliable with restrictions), and category 3 (score <11; not reliable).

### 2.5 Data Analysis

For inferential purposes, a fixed-effect model was used. Standardized mean difference (SMD) was chosen for consolidating the statistical data. The data of each study was analyzed using the odds ratio (OR) and 95% confidence interval. Forest plots and the I^2^ index were calculated to detect heterogeneity. Thresholds of I^2^ were in line with Cochrane recommendations: 0%–40% (“might not be important”), 30%–60% (“may represent moderate heterogeneity”), 50%–90% (“may represent substantial heterogeneity”), and 75%–100% (“considerable heterogeneity”). The importance of the I^2^ value was interpreted alongside the *p*-value from the Chi-squared test. Outcome analysis was performed based on the exposure duration (24 h and >24 h). A funnel plot was used to assess the presence of publication bias for small-study effects. The statistical significance (*p* < 0.05) is based on the data provided in the original publications. MedCalc Version 19.6.1 software was used to perform all the computations.

## 3 Results

### 3.1 Systematic overview

Search results were based on the different combinations of keywords (“Guggulsterone”, “*Commiphora mukul*”, and “*Commiphora wightii*” in combination with “HCC”, “Hepatocellular carcinoma”, “Liver cancer”, “Cancer”, “Cirrhosis”, and “Fibrosis”). The search results fetched 55,280 records from seven different databases viz. 1675 from Pubmed, 8487 from PMC, 38 from Cochrane library, 4 from ctri.gov.in, 40185 from Google Scholar, 3486 from Science Direct and 1405 from Scopus.

Out of the 55,280 articles, the 716 articles obtained from the keyword “Guggulsterone and Cancer” were used further for the study, and the remaining articles were excluded. A manual bibliographic search was also done and 138 articles were screened through it. The resulting 854 articles (716 + 138) were later subjected to duplicate exclusion. 4 duplicates were found and excluded and the remaining 850 articles were subjected to title and abstract screening. Out of the 850 articles, 725 articles were found to be irrelevant to the subject in focus and did not fulfill the inclusion criteria and were thus, excluded. The remaining 125 articles were assessed for eligibility, and 87 of them were found to be eligible for analysis. From a pool of 87 articles, 9 articles reported *in vivo* studies (Supplementary Table S1), 38 studies were based on the use of Guggulsterone only as an FXR antagonist, and 40 studies were based on the therapeutic implications of Guggulsterone in various cancer cells lines. The *in vivo* studies and studies using Guggulsterone as an FXR antagonist were excluded. Out of the 40 remaining studies, 17 studies were again excluded as they differ from the objective and only 23 articles were included in the study as they met the following inclusion criteria: i) apoptosis induced by Guggulsterone tested *in vitro* and/or ii) Guggulsterone-induced cell cycle arrest. The articles providing a detailed description of the experimental design and findings were finally included in the quantitative analysis.

**FIGURE 3:**
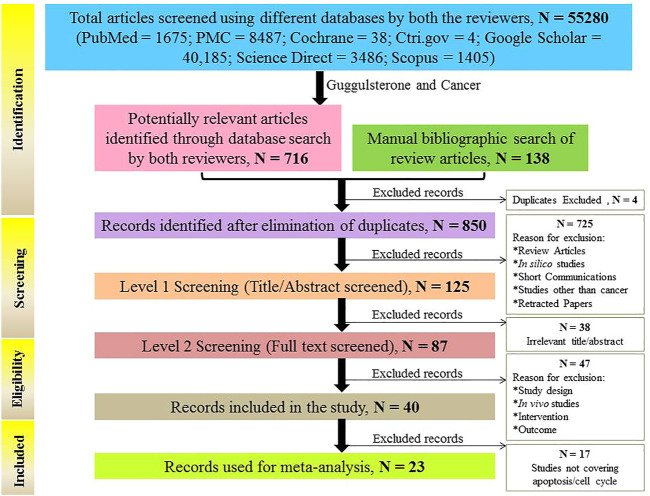
Flow chart explaining the selection process of studies (*in vitro*) included in the meta-analysis.

All of the articles reported the protective role of Guggulsterone against various cancer types *in vitro* at different doses and durations. The PRISMA flow diagram describing the search strategy and study selection process is available in Figure 3.

### 3.2 Meta-analysis

#### 3.2.1 Study characteristics

The selected studies were classified based on the i) type of cancer, ii) cell lines used iii) dose of Guggulsterone used in the study and duration of its treatment, iv) type of assay conducted to determine the apoptotic effect v) outcomes and pathway of drug action. The main characteristics of the studies included in the meta-analysis are summarised in Table 1. All the selected studies reported the apoptotic effect of Guggulsterone in various cancer cells at different time points and doses.

**TABLE 1:** Main characteristics of the 23 studies included in the meta-analysis related to the role of Guggulsterone and apoptosis induction in cancer cells.

S. No.	Study	Cancer	Cell line	Guggulsterone dose/Duration of treatment	Assay used	Apoptosis effect		Gene regulation	Pathway of action
						Control	Guggulsterone intervention		
1	Macha et al. (2010)	Head and Neck squamous cell carcinoma	SCC4	50μM/24,48hrs	Annexin V/PI	GGS@0 h:5%	GGS@24 h: 40%	↓ Bcl-2, xIAP, Mcl1, survivin, cyclin D1 and c-myc	Intrinsic mitochondrial pathway by releasing Bad from its inhibitory action
							GGS@48 h: 60%	↑ caspase 9, caspase 8, and caspase 3 leading to the cleavage of PARP	
2	De Gottardi et al. (2006)	Oesophageal adenocarcinoma	CP-18821	20μM/48hrs	Flow cytometry	6.9% ± 0.8%	15.1% ± 3.5%	↑ caspase 3	Guggulsterone, an FXR antagonist induced apoptosis by targeting and inhibiting FXR
								↓ cyclin D1 and c-myc	
3	Yamada et al. (2010)	Gut derived Adenocarcinoma	Bic-1	5μM/24hrs	Flow cytometry	Sub G1:1.9%	Sub G1: 3.4%	↓ bile acid-induced CdX2 expression	Guggulsterone targets and inhibits CdX2
						G1/G0:68.7%	G1/G0: 66.4%		
						S:19.2%	S: 18.4%		
						G2/M:10.2%	G2/M: 11.8%		
4	Lv et al. (2021)	Gastric cancer	SGC-7901	50,75μM/24hrs	Annexin V/PI	Early apoptosis: 4.08%	50μM/Early apoptosis: 4.24%	↓ VEGF, TGF-*β*1 and Bcl-2	Mitochondrial dependent apoptotic pathway
							50μM/Late apoptosis: 11.8%		
						Late apoptosis: 4.61%	75μM/Early apoptosis: 7.32%	↑ TNF-*α,* caspase-3, Bax	
							75μM/Late apoptosis: 19.8%		
5	Ahn et al. (2012)	Pancreatic cancer	MiaPaCa-2, Panc-1	5,10,20,30μM/48hrs	Annexin V/PI	MiaPaca-2: ∼3%	2μM/MiaPaca-2: 12%	↓NF-kB, Akt and BcL-2	AKT and NF-kB signalling Pathway
							10μM/MiaPaca-2: 18%	↑c-Jun NH_2_-terminal kinase and Bax	
							20μM/MiaPaca-2: 38%		
							30μM/MiaPaca-2: 45%		
						Panc-1: 7%	2μM/Panc-1: 9%		
							10μM/Panc-1:12%		
							20μM/Panc-1: 35%		
							30μM/Panc-1: 45%		
6	Zhong et al. (2015a)	Cholangiocarcinoma	HuCC-T1, RBE	1,2,5,10,20μM/24hrs	Annexin V/FITC	HuCC-T1: 3%	1μM/HuCC-T1: 10%	↑caspase3, caspase 8 and caspase 9	ROS/JNK Signaling pathway mediated apoptosis
							2μM/HuCC-T1: 17%		
							5μM/HuCC-T1: 25%		
							10μM/HuCC-T1:32%		
							20μM/HuCC-T1: 44%		
						RBE: 4%	1μM/RBE: 10%		
							2μM/RBE: 15%		
							5μM/RBE: 24%		
							10μM/RBE:36%		
							20μM/RBE: 55%		
7	An et al. (2009)	Colon cancer	HT-29	25,50μM/48hrs, 72hrs	Annexin V-FITC	48 h: 3%	25μM/48 h:12%	↓ caspase-9, ↑ caspase-3, caspase-8	mitochondria-dependent pathway and the extrinsic pathway of apoptosis
							50μM/48 h:14%	↓ cIAP-1, cIAP-2, tBid, and Bcl-2	
						72 h: 4%	25μM/72 h:32%	↑ truncated Bid, Fas, p-JNK, and p-c-Jun	
							50μM/72 h: 37%		
8	Leeman-Neill et al. (2009)	Head and Neck squamous cell carcinoma	UM-22b, 1483	10,20μM/24hrs	Flow cytometry	1483: 39%	10μM/1483:45%	↓ pro-caspase 3	STAT3 signaling
							20μM/1483:60%	↓ phospho-tyrosine and total STAT-3	
						UM-22B: 37%	10μM/UM-22B: 40%	↓ HIF-1a	
							20μM/UM-22B: 48%		
9	Leo et al. (2019)	Colorectal cancer	HCT116	50μM/24,48 h	Annexin V/PI	24 h: 5%	24 h: 25%	↑ p53 protein, ↓ NF-kB, Bcl-2, cIAP-1, and survivin	p53 mediated intrinsic apoptotic pathway; NF-kB signalling pathway
						48 h: 10%	48 h: 90%		
10	Chen et al. (2021)	Bladder cancer	T24, TSGH8301	40,60μM/24hrs	Annexin V-FITC	T24: 7%	40μM/T24: 8.5%	↓ cleaved caspase-3 and cleaved PARP	mTOR-Akt signalling pathway and autophagic pathway
							60 μM/T24: 8%		
						TSGH8301: 3.8%	40μM/TSGH8301:5.8%		
							60 µM/TSGH8301: 6%		
11	Dixit et al. (2013)	Glioblastoma	A172, U87, T98G	30μM/48hrs	Tunel Assay	A172: 2%	30μMSANT-1/A172:4%	↓Ras and NF-kB activity, Apoptosis via intrinsic apoptotic mechanism,↑ERK and ↑ Caspase-9 activation	Ras and NF-kB signaling; mitochondrial apoptotic pathway
							30μMGUG/A172: 18%		
							30μMGUG+30μMSANT-1/A172: 49%		
						T98G: 5%	30μMSANT-1/T98G:12%		
							30μMGUG/T98G: 25%		
							30μMGUG+30μMSANT-1/T98G: 44%		
						U87: 2.5%	30μMSANT-1/U87:3%		
							30μMGUGG/U87: 20%		
							30μMGUG+30μMSANT-1/U87: 42%		
12	Choudhuri et al. (2011)	Pancreatic cancer	PC-Sw	50μM/24hrs	Flow cytometry	Go/G1: 34%	51%	Inhibited radiation induced NF-κB activation and enhanced radiosensitivity	NF-κB signalling and involvement of IGF1-Rβ
						S: 44%	36%	↓ERα and IGF1-Rβ	
						G2/M: 22	12		
13	Moon et al. (2011)	Hepatocellular Carcinoma	Hep3B	30µM/24hrs	Annexin V Assay	2.6%	GGS:15.2%	DR5↑ and CHOP↑ (RTPCR)	Induction of CHOP-dependent DR5
							TRAIL:8.7%	GGS + TRAIL: Caspase-8, caspase-9, caspase-3↑ (Western Blotting)	
							GGS + TRAIL:31.3%		
14	Shishodia et al. (2007)	Histiocytic leukemia	U937	10μM/24,48hrs	Annexin V Assay	6%	GGS@24 h: 38%	Bfl-1/A1, XIAP, cFLIP, Bcl-2, BclXL, and survivin↓ (Western Blotting)	Activation of JNK, suppression of Akt, and down-regulation of anti-apoptotic protein expression
							GGS@48 h: 87%	COX-2 and c-myc↓ (Western Blotting)	
								COX-2, IL-1β, IL-6, and TNF↓ (RTPCR)	
								Caspase activation, cytochrome c release and PARP cleavage (Western Blotting)	
15	Macha et al. (2013)	Pancreatic cancer	CD18/HPAF, Capan1	50μM/24hrs	Annexin V staining and flow cytometry	Capan1: 2% CD18/HPAF: 3.1%	GGS: 37%	xIAP, Bcl2, Cyclin D1↓	JAK/STAT and Src/FAK signaling
							GGS: 32%	Cleaved Caspase-3↑(Western Blotting)	
								Cyclin D1, Abcl2, Survivin↓	
								BAD and Bax↑	
								No change in expression of Bak (RTPCR)	
16	Zhong et al. (2015b)	Cholangiocarcinoma	Sk-ChA-1, Mz-ChA-1	20,40,60μM/24, 48, 72hrs	Flow cytometry	Sk-ChA-1: 0% Mz-ChA-1: 0%	20μM/24h/Mz-ChA-1: 8%	Survivin, Bcl-2↓	Caspase-dependent apoptosis and downregulation of survivin and Bcl-2 expression
							40μM/24h/Mz-ChA-1: 15%	Bax remained constant (Western Blotting)	
							60μM/24h/Mz-ChA-1: 21%	Caspases-8, - 9 and -3↑ (colorimetric assay)	
							20μM/24h/Sk-ChA-1: 17%		
							40μM/24h/Sk-ChA-1: 18%		
							60μM/24h/Sk-ChA-1: 33%		
							20μM/48h/Mz-ChA-1: 21%		
							40μM/48h/Mz-ChA-1: 25%		
							60μM/48h/Mz-ChA-1: 29%		
							20μM/48h/Sk-ChA-1: 29%		
							40μM/48h/Sk-ChA-1: 30%		
							60μM/48h/Mz-ChA-1: 35%		
							20μM/72h/Mz-ChA-1: 45%		
							40μM/72h/Mz-ChA-1: 48%		
							60μM/72h/Mz-ChA-1: 51%		
							20μM/72h/Sk-ChA-1: 39%		
							40μM/72h/Sk-ChA-1: 45%		
							60μM/72h/Mz-ChA-1: 56%		
17	Samudio et al. (2015)	Acute myeloid leukemia	HL60, U937	10,15,20μM/72hrs	Annexin V staining	HL60: 9%	HL60/10μM/16-Dehydroprogesterone: 18%	Cleaved caspase-3↑ (Western Blotting)	Induction of apoptosis and differentiation
						U937: 9%	HL60/15μM/16-Dehydroprogesterone: 30%		
							HL60/20μM/16-Dehydroprogesterone: 42%		
							HL60/10μM/cGGS: 35%		
							HL60/15μM/cGGS: 60%		
							HL60/20μM/cGGS: 70%		
							HL60/10μM/tGGS: 15%		
							HL60/15μM/tGGS: 32%		
							HL60/20μM/tGGS: 50%		
							U937/10μM/16-Dehydroprogesterone: 20%		
							U937/15μM/16-Dehydroprogesterone: 28%		
							U937/20μM/16-Dehydroprogesterone: 30%		
							U937/10μM/cGGS: 18%		
							U937/15μM/cGGS: 30%		
							U937/20μM/cGGS: 62%		
							U937/10μM/tGGS: 20%		
							U937/15μM/tGGS: 21%		
							U937/20μM/tGGS: 39%		
18	Singh et al. (2005)	Prostate	PC-3	10,20μM/24hrs	Flow cytometry	57 ± 1%	10 μM: 59 ± 1%	Bax↑, Bak (8–12 h) ↑ but declined thereafter, Bcl (2–12 h) ↑, Bcl (16–24 h)↓	Caspase-dependent apoptosis
		Cancer					20 μM: 61 ± 1%	Cleaved caspase-3, -8 and -9↑ (Western Blotting)	
19	Tian et al. (2021)	Non-small cell lung cancer	A549, H1975	10,20,40μM/48hrs	Flow cytometry	A549: 68%	(G0/G1 phase)	PD-L1↑ (RTPCR)	Induction of PD-L1 upregulation partly mediated by FXR, Akt and Erk1/2 signaling pathways
			LLC: Normal cell line			H1975: 46%	A549/GGS10 μM: 72%		
						LLC: 42%	A549/GGS20 μM: 74%		
							A549/GGS40 μM: 78%		
							H1975/GGS10 μM: 54%		
							H1975/GGS20 μM: 70%		
							H1975/GGS40 μM: 72%		
							LLC/GGS10μM: 52%		
							LLC/GGS20μM: 54%		
							LLC/GGS40μM: 64%		
20	Shi et al. (2015)	Hepatocellular carcinoma	HepG2	50,75μM/24hrs	Annexin	5.18% ± 1.74	50 μM: 24.91% ± 2.41%	Bax↑, Bcl-2↓ (RTPCR)	Induction of apoptosis through intrinsic mitochondrial pathway
					V-FITC and PI double staining		75 μM: 53.03% ± 2.28%		
21	Xu et al. (2011)	Breast cancer	MCF-7/DOX	10μM/48hrs	Annexin V/FITC and PI double-staining	0.72 ± 0.09	GGS: 2.42 ± 1.03%	GGS has no influence on the expression of MRP1 protein	Reversal of Multidrug Resistance
				DOX: 10μΜ			DOX: 4.05 ± 0.61%		
							GGS + DOX: 24.91 ± 4.57%		
22	Xu et al. (2017)	Hepatocarcinoma	PLC/PRF/5R	50μM/48hrs	Annexin V-FITC/PI	1.5%	GGS: 8.72%	Cox-2, Pgp↓	Cox-2/P-gp dependent pathway
				Verapamil:10 μM			V: 9.26%		
				Celecoxib:10 μM			C: 9.08%		
				Doxorubicin: 10 μM			D: 13.15%		
							D + GGS: 20.16%		
							D + V: 24.47%		
							D + C: 21.19%		
23	Yamada et al. (2014)	Esophageal Adenocarcinoma	OE19	25,50μM/24 h	Flow Cytometric Analysis	6%	(Sub G1 phase)	Cleaved caspase-3, PARP↑	Suppression of CDX2 and COX-2 expression
							25 μM: 18%		
							50 μM: 30%		

#### 3.2.2 Analysis based on the apoptotic effect of guggulsterone

The selected 23 studies reporting the apoptotic effect of Guggulsterone in various cancer cell lines were eligible for meta-analysis. The percentage apoptosis compared to control was used as the primary outcome measure. The studies were grouped into 2 sub-sets based on the time point viz. 24 h and >24 h. In the first subset, the studies with the therapeutic role of Guggulsterone in the induction of apoptosis in cells treated for 24 h duration as compared to untreated cells were considered. Among the 23 studies, 11 studies reported the apoptotic effect of Guggulsterone at 24 h (Singh et al., 2005; Leeman-Neill et al., 2009; Yamada et al., 2010; Choudhuri et al., 2011; Moon et al., 2011; Macha et al., 2013; Yamada et al., 2014; Shi et al., 2015; Zhong et al., 2015a; Chen et al., 2021; Lv et al., 2021). The second subset included the Guggulsterone-induced apoptosis in the treated cancer cells for >24 h. 7 of 23 studies reported the apoptotic effect of Guggulsterone at t > 24 h i.e. either at 48 h or 72 h. 6 of these 7 studies reported the effect of Guggulsterone at 48 h (De Gottardi et al., 2006; Xu et al., 2011; Ahn et al., 2012; Dixit et al., 2013; Xu et al., 2017; Tian et al., 2021) and 1 study used the treatment for 72 h (Samudio et al., 2015). 5 of 23 studies reported the apoptotic effect at multiple time points i.e. at 24 and 48 h (Shishodia et al., 2007; Macha et al., 2010; Leo et al., 2019), at 48 and 72 h (An et al., 2009), and at 24, 48 and 72 h (Zhong et al., 2015b).

##### 3.2.2.1 Subset-1: Apoptotic effect of guggulsterone after 24 h of treatment

**FIGURE 4:**
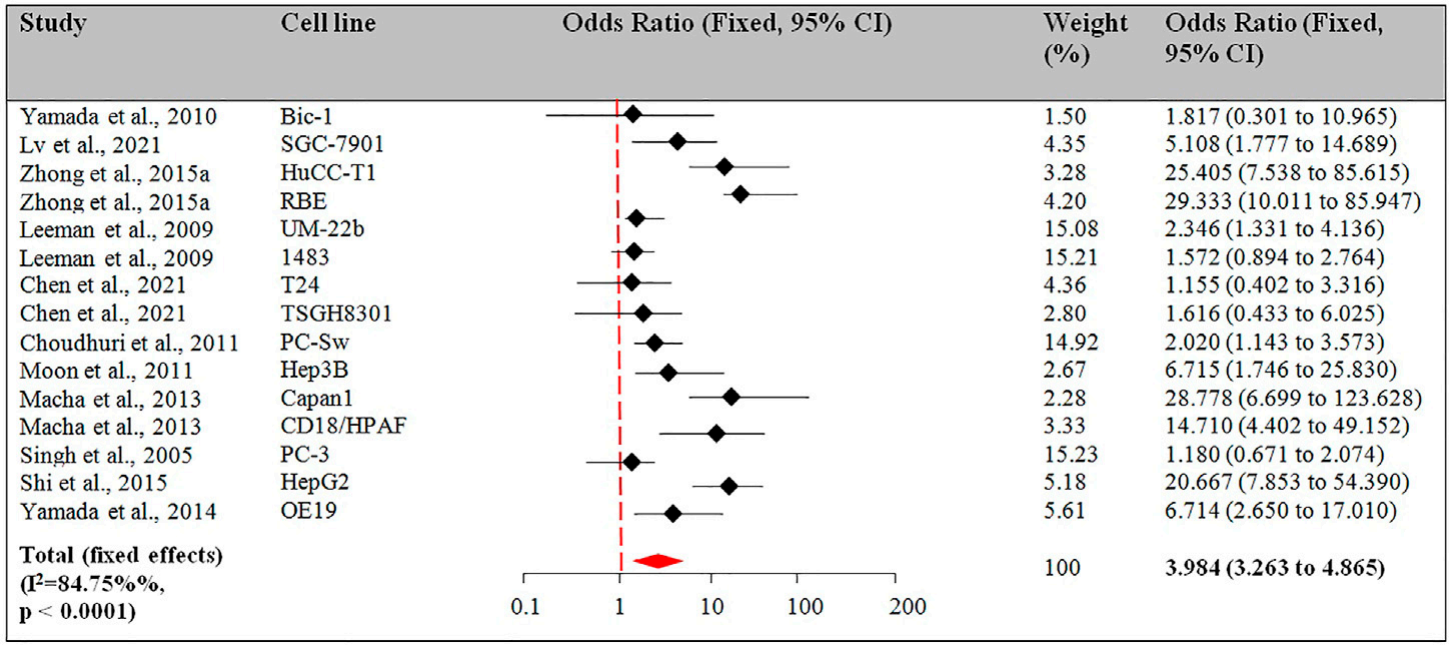
Guggulsterone (t = 24 h) *versus* control odds ratio in cancer cells. Fixed effects meta-analysis was performed for the outcomes to compare data for Guggulsterone (t = 24 h) *versus* control and the odds ratio was determined.

Among the 23 studies, there were 11 studies for this outcome. 4 of the 11 studies reported the apoptotic effect in more than 1 cell line (Leeman-Neill et al., 2009; Macha et al., 2013; Zhong et al., 2015a; Chen et al., 2021). The pooled OR for the fixed model effect was 3.984 (CI 3.263 to 4.865, *p* < 0.001). Figure 4 describes the results from the fixed-effects model combining the OR for the apoptotic effect of Guggulsterone exposed for 24 h treatment.

##### 3.2.2.2 Subset-2: Apoptotic effect of guggulsterone after t > 24 h of treatment

**FIGURE 5:**
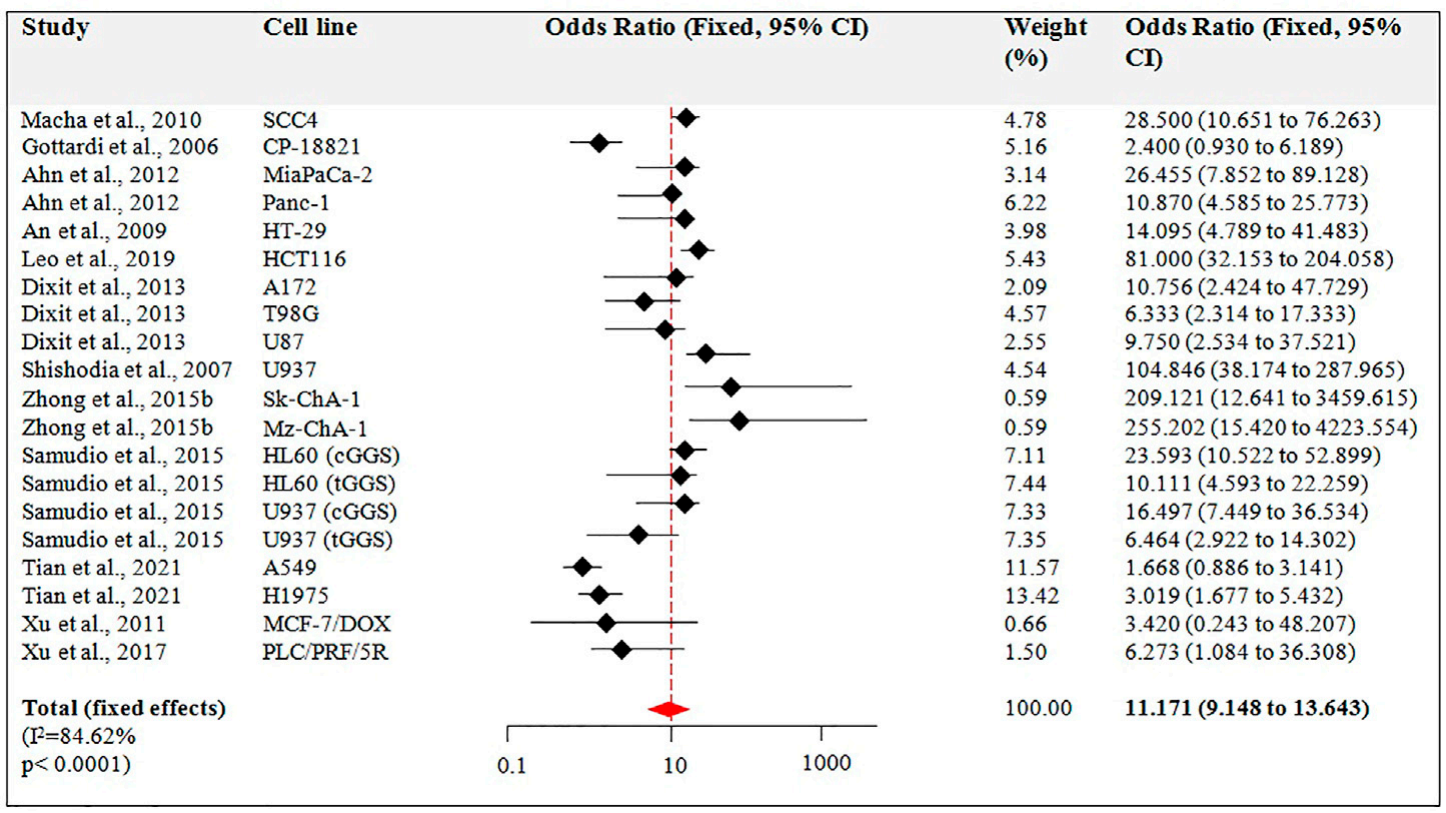
Guggulsterone (t > 24 h) versus control odds ratio in cancer cells. Fixed effects meta-analysis was performed for the outcomes to compare data for Guggulsterone (t > 24 h) versus control and the odds ratio was determined.

Figure 5 describes the results from the fixed-effects model combining the OR for the apoptotic effect of Guggulsterone exposed for a time >24 h. There were 12 studies for this outcome. Among these 12 studies, 5 studies used multiple cell lines to report the apoptotic effect of Guggulsterone (Ahn et al., 2012; Dixit et al., 2013; Samudio et al., 2015; Zhong et al., 2015b; Tian et al., 2021). The OR for the association varied from 9.148 to 13.643 across studies. Overall, the combined OR showed significant apoptosis in the cancer cells treated with Guggulsterone as compared to the control (OR: 11.171, 95% CI, *p* < 0.001).

#### 3.2.3 Subgroup analyses based on cancer type

**FIGURE 6:**
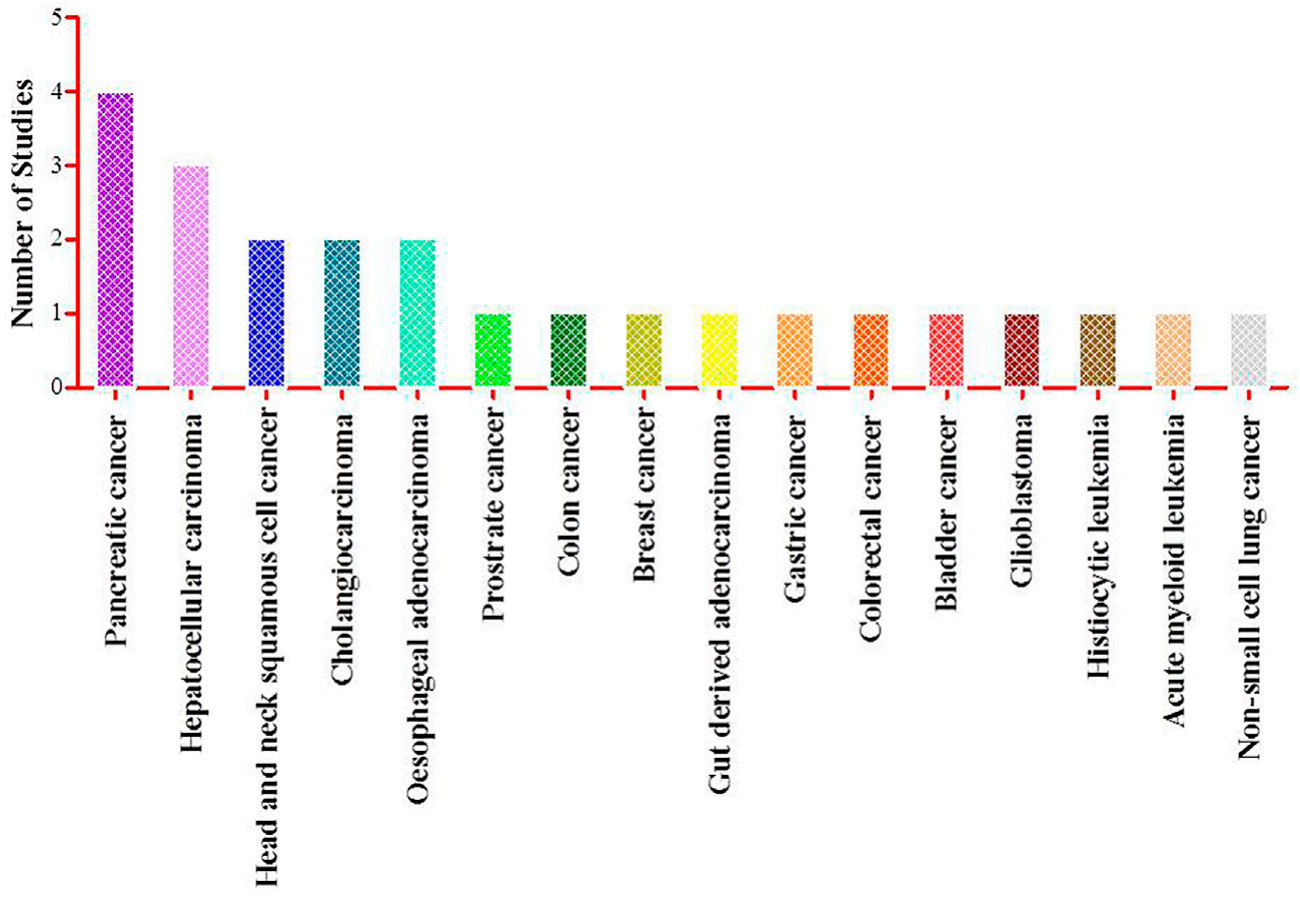
Graphical representation of different types of cancer studied in 23 selected articles.

The included studies involved the effect of Guggulsterone in the treatment of various cancers. Three studies were on pancreatic cancer (MiaPaCa-2, Panc-1, PC-Sw, CD18/HPAF, Capan1, PC-3) (Choudhuri et al., 2011; Ahn et al., 2012; Macha et al., 2013), three on hepatocellular carcinoma (Hep3B, HepG2, PLC/PRF/5R) (Moon et al., 2011; Xu et al., 2011; Shi et al., 2015), two studies each on head and neck squamous cell carcinoma (SCC4, UM-22b, 1483) (Leeman-Neill et al., 2009; Macha et al., 2010), cholangiocarcinoma (HuCC-T1, RBE, Sk-ChA-1, Mz-ChA-1) (Zhong et al., 2015a; Zhong et al., 2015b) and oesophageal adenocarcinoma (CP-18821, OE19) (De Gottardi et al., 2006; Yamada et al., 2010) and one study each on prostrate cancer (PC-3) (Singh et al., 2005), colon cancer (HT-29) (An et al., 2009), breast cancer (MCF7/DOX) (Xu et al., 2011), gut derived adenocarcinoma (Bic-1) (Yamada et al., 2010), gastric cancer (SGC-7901) (Lv et al., 2021), colorectal cancer (HCT116) (Leo et al., 2019), bladder cancer (T24, TSGH8301) (Chen et al., 2021), glioblastoma (A172, U87MG, T98G) (Dixit et al., 2013), histiocytic leukemia (U937) (Shishodia et al., 2007), acute myeloid leukemia (HL60, U937) (Samudio et al., 2015) and non-small cell lung cancer (A549, H1975) (Tian et al., 2021). All the studies reported that Guggulsterone exerts its anticancer effects by inducing apoptotic pathways, inhibiting cell proliferation, and regulating the expression of genes involved in apoptosis. Figure 6 shows the graphical representation of this data.

#### 3.2.4 Subgroup analyses based on guggulsterone dose

**FIGURE 7:**
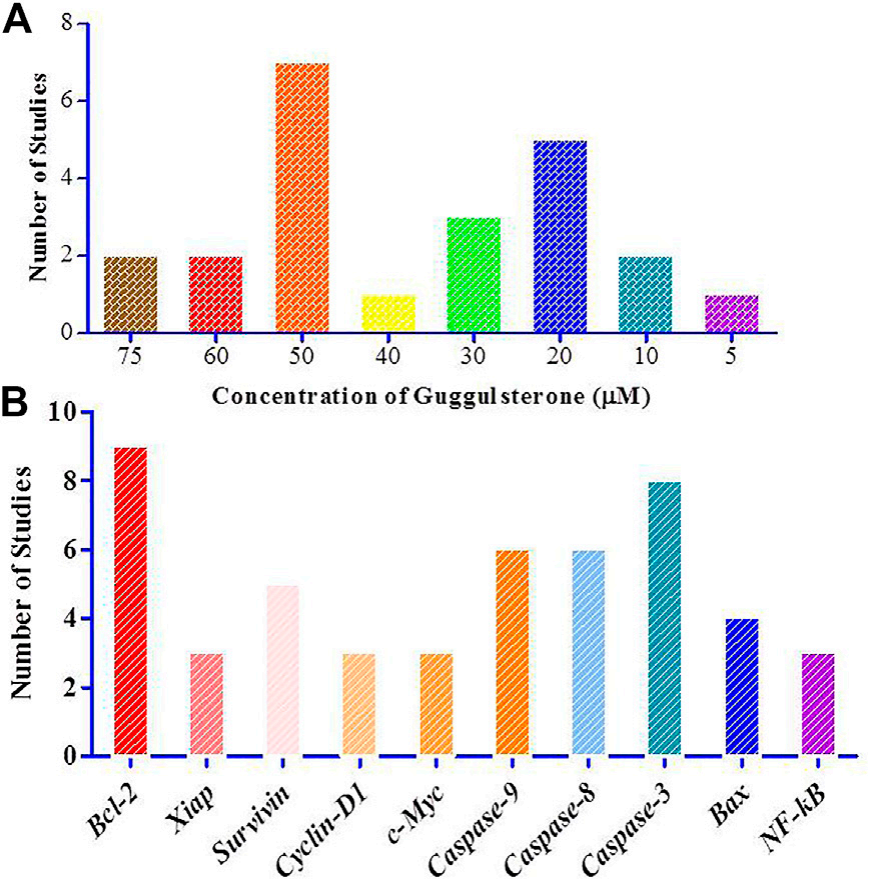
Graphical representation of the study characteristics and the observations **(A)**. Concentration of Guggulsterone used for the treatment of the various cancer *in vitro* in selected 23 studies; **(B)**. Number of studies that have shown the modulation of these major genes.

8 out of 23 studies used Guggulsterone at a dose greater than 20 μM (Singh et al., 2005; De Gottardi et al., 2006; Shishodia et al., 2007; Leeman-Neill et al., 2009; Yamada et al., 2010; Xu et al., 2011; Samudio et al., 2015; Zhong et al., 2015b). The highest doses used were 75 μM (n = 2) (Shi et al., 2015; Lv et al., 2021), 60 μM (n = 2) (Zhong et al., 2015b; Chen et al., 2021), 50 μM (n = 7) (An et al., 2009; Macha et al., 2010; Choudhuri et al., 2011; Macha et al., 2013; Yamada et al., 2014; Xu et al., 2017; Leo et al., 2019), 40 μM (n = 1) (Tian et al., 2021) and 30 μM (n = 3) (Moon et al., 2011; Dixit et al., 2013; Tian et al., 2021). 20μM concentration of Guggulsterone was used in 5 studies (Singh et al., 2005; De Gottardi et al., 2006; Leeman-Neill et al., 2009; Zhong et al., 2015a; Samudio et al., 2015) and 3 studies used the intervention at a dose less than 20 μM (Shishodia et al., 2007; Macha et al., 2010; Yamada et al., 2010). The doses used were 10 μM (n = 2) (Shishodia et al., 2007; Macha et al., 2010) and 5 μM (n = 1) (Yamada et al., 2010). The data has been graphically presented in Figure 7A.

#### 3.2.5 Subgroup analyses of guggulsterone treatment effects

Treatment of these cell lines with Guggulsterone brought alterations in the status of cancer-critical genes. Significant upregulation in the level of caspase-9 was discussed in 7 studies (Shishodia et al., 2007; An et al., 2009; Macha et al., 2010; Moon et al., 2011; Dixit et al., 2013; Zhong et al., 2015a; Zhong et al., 2015b). Further, 5 studies reported the upregulation of caspase 8 (Shishodia et al., 2007; Macha et al., 2010; Moon et al., 2011; Zhong et al., 2015a; Zhong et al., 2015b) and 8 studies discussed the increased expression of caspase-3 (De Gottardi et al., 2006; Shishodia et al., 2007; Macha et al., 2010; Moon et al., 2011; Samudio et al., 2015; Zhong et al., 2015a; Zhong et al., 2015b; Lv et al., 2021). 4 studies reported the upregulation of Bax as observed and analyzed by qRTPCR and western blotting (Singh et al., 2005; Ahn et al., 2012; Shi et al., 2015; Lv et al., 2021). In cancer cells, Guggulsterone decreased the expression of Bcl-2 (n = 9) (Singh et al., 2005; Shishodia et al., 2007; An et al., 2009; Yamada et al., 2010; Ahn et al., 2012; Macha et al., 2013; Zhong et al., 2015b; Shi et al., 2015; Leo et al., 2019), xiAP (n = 3) (Shishodia et al., 2007; Macha et al., 2010; Macha et al., 2013), survivin (n = 5) (Shishodia et al., 2007; Macha et al., 2010; Macha et al., 2013; Leo et al., 2019; Zhong et al., 2015b), cyclin D (n = 3) (De Gottardi et al., 2006; Yamada et al., 2010; Macha et al., 2013), c-myc (n = 3) (De Gottardi et al., 2006; Yamada et al., 2010; Macha et al., 2013) and NF-κβ (n = 3) (Ahn et al., 2012; Dixit et al., 2013; Leo et al., 2019). The levels of JNK, ciAP-1, cleaved caspase 3, and cox-2 were also altered when subjected to Guggulsterone treatment (Figure 7B).

### 3.3 Heterogeneity test and publication bias

The scale of heterogeneity not imputable to the sampling error was determined by the I^2^ value. When the cells were exposed to the treatment for 24 h, the total amount of heterogeneity was considerable (I^2^ = 84.75%, *p* < 0.0001). Treatment with Guggulsterone for time >24 h also showed considerable heterogeneity (I^2^ = 84.62%, *p* < 0.0001). Visual inspection of the funnel plot showed some asymmetry in both cases (Supplementary Figure S1A and S1B). Supplementary Table S2 summarizes the ToxR reliability assessment score for the individual *in vitro* toxicity studies. Of the 23 evaluated studies, all the studies were found to be “Reliable Without Restriction”. Even, checking the weighted scores (red scores) did not revise the results.

## 4 Discussion

Guggulsterone is a plant sterol present in the gum resin of the Commiphora species and is known for its pleiotropic effects in the treatment of multiple human diseases (Owsley and Chiang, 2003; Bhat et al., 2017; Kunnumakkara et al., 2018). It has been found to induce apoptosis in many cancer types via the modulation of apoptotic proteins and survival signaling pathways (Lv et al., 2021). In the present study, we summarized all (until June 2021) *in vitro* experiments investigating the apoptotic effect of Guggulsterone on cancer cells. In total, 23 *in vitro* studies were of interest and were thoroughly analyzed. Of these 23 studies, 8 studies used “guggulsterone” and 12 studies used “z-guggulsterone” for their research. However, the remaining 3 studies used both the forms of guggulsterone: one study used both compounds in all the tests conducted (Samudio et al., 2015), one used an even mixture (Leeman-Neill et al., 2009), and in the third study, both form were initially used for comparsion and based upon the results, “e” form was selected for further research (Chen et al., 2021). 23 studies included multiple cancer types, cell lines, the dose of Guggulsterone and time points, regulation of various genes, and different pathways of action thereby enhancing the relevance of these results.

Our meta-analysis confirms and strengthens previous evidence that Guggulsterone (5–75 μM) is effective in inducing apoptosis in cancer cell lines. In particular, cancer cells treated with Guggulsterone for 24 h showed an odds ratio of 3.984 (CI 3.263 to 4.865, *p* < 0.001) compared to the control. When cells were exposed to different concentrations of Guggulsterone for t˃24h, the odds ratio reported was 11.171 (CI 9.148 to 13.643, *p* < 0.001). This shows that Guggulsterone induces apoptosis in a time-dependent manner and the results are consistent with the previous reports (Shishodia et al., 2007; An et al., 2009; Zhong et al., 2015b).

Bioactive compounds have several action mechanisms involved in the induction of cancer cell apoptosis. In tumor cells, they function via modulating the extrinsic and intrinsic pathways of apoptosis, channeling important cell signaling pathways such as mTOR-AKT, Ras/NF-κβ, ROS/JNK, JAK/STAT, and Src/Fak and regulating gene expression (Huang et al., 2018; Gupta et al., 2021; Gupta et al., 2022a; Gupta et al., 2022b; Gupta et al., 2023). Treatment with Guggulsterone also altered the expression of different gene families which collaborate in the induction of apoptosis. For instance, in the present study, the levels of the members of the caspase family, the conserved cysteine aspartic-specific proteases which are considered crucial in apoptosis induction (Brentnall et al., 2013), were found to be increased. Significant upregulation in the level of caspase-9, the initiator caspase important in the formation of apoptosome complex in the mitochondrial pathway of apoptosis (Li et al., 2017), was observed. Further, caspase-8 and caspase-3 were also found to be upregulated in the studies. Mechanistically, caspase 8 either directly induces the apoptotic pathway or activates caspase-3. Another pro-apoptotic protein Bax (a BCL-2 family protein) was found to be upregulated in the analyzed studies. Bax is involved in the mitochondrial pathway of apoptosis and stimulates the release of cytochrome c from the mitochondria thereby promoting the apoptosis of cancer cells (Shi et al., 2015; Lv et al., 2021). Further, in the cancer cells, Guggulsterone decreased the expression of anti-apoptotic protein thereby leading to increased apoptosis viz. Bcl-2; xiAP (apoptotic inhibitor). Survivin, another identified mammalian inhibitor of apoptosis, was found to be decreased in a few studies. A reduction in the expression of genes and proteins involved in cancer cell proliferation was also observed in various studies (cyclin D1, c-myc, NF-κβ). The levels of other genes involved in the apoptotic pathways viz. JNK, ciAP-1, cleaved caspase 3, and cox-2 were also altered when subjected to Guggulsterone treatment.

Our study, being the first of its kind, is characterized by strengths like extensive literature search and meta-analysis, and highlighting promising results for Guggulsterone, it is plagued with shortcomings as well. Above all is the high heterogeneity between the studies, which is probably due to a limited number of studies (leading to multiple cancer types and cells, different apoptotic assays, and different study designs). Although all the databases are screened expansively and carefully, still the studies on a particular cancer type are very less. Therefore, a generalizable interpretation was affected by high study variability. In general, to overcome heterogeneity, it is advised to explore the heterogeneity by conducting subgroup analyses, changing the effect measure, and excluding studies from the meta-analysis based on their results. Next, the funnel plot revealed publication bias, which is also due to the limited number of articles and therefore, suggests more quality research papers on this molecule.

## 5 Conclusion and future directions

Meta-analysis and systematic review of basic research offer collective and critical insights into the current state of knowledge in the respective field. In this manuscript, we have used a meta-analytic approach on *in vitro* studies to evaluate the apoptotic effects of Guggulsterone against various cancer types and to the best of our knowledge, it is the first meta-analysis of this kind. Our current findings are promising and highlight the importance of Guggulsterone in cancer management, which may direct future research. However, data is still insufficient to establish the effect of Guggulsterone on various cancer types. The statistical evaluation shows considerable heterogeneity and publication bias among the studies, which, however, does not invalidate the efficacy of Guggulsterone as there is a significant difference in the treated and control groups. Existing studies establish the role of Guggulsterone as a potential candidate; alongside, provide a rationale for further pre-clinical and clinical evaluation to develop this molecule as an anticancer agent. Additionally, the isolation and chemical synthesis of guggulsterone is an exorbitant process, which might be the reason that a limited number of studies are utilizing it. Therefore, more research should be done for the economical chemical synthesis of this very important molecule.

## Data Availability

The original contributions presented in the study are included in the article/Supplementary Material, further inquiries can be directed to the corresponding author.
