# Supplementary material for: Anti-cancer activity of guggulsterone by modulating apoptotic markers: a systematic review and meta-analysis
Source: Front Pharmacol. 2023 May 2;14:1155163. doi: 10.3389/fphar.2023.1155163 (PMC10185795; doi:10.3389/fphar.2023.1155163)
Supplement: Supplementary file 2 [file DataSheet1.docx]

**SUPPLEMENATRY MATERIAL**

**Anti-Cancer Activity of Guggulsterone by Modulating Apoptotic Markers: A Systematic Review and Meta-Analysis Study**

Meenakshi Gupta^1^, Deepti Singh^2^, Shruti Rastogi^1,3^, Hifzur R.Siddique^2^, Ajaz Ahmad^4^, Mohammad Sikander^5^, Maryam Sarwat^1*^

^1^Amity Institute of Pharmacy, Amity University, Sector-125, Noida-201301, Uttar Pradesh, India

^2^Molecular Cancer Genetics & Translational Research Lab, Section of Genetics, Department of Zoology, Aligarh Muslim University, Aligarh 202002, India

^3^Indian Pharmacopoeia Commission, Ministry of Health & Family Welfare, Government of India, Ghaziabad-201002, Uttar Pradesh, India

^4^Department of Clinical Pharmacy, College of Pharmacy King Saud University, Riyadh 11451, Saudi Arabia

^5^Department of Immunology and Microbiology, Biomedical Research, The University of Texas, Rio Grande Valley, 5300 North L Street, McAllen TX 78504, United States

**Running title:** Meta-Analysis on Guggulsterone

*Corresponding author:

**Maryam Sarwat**

Amity Institute of Pharmacy,

Amity University

Sector-125, Noida-201301,

Uttar Pradesh, India.

Tel.: +91-9990373875

E-mail: [msarwat@amity.edu](mailto:msarwat@amity.edu), [maryam21_7@yahoo.com](mailto:maryam21_7@yahoo.com)


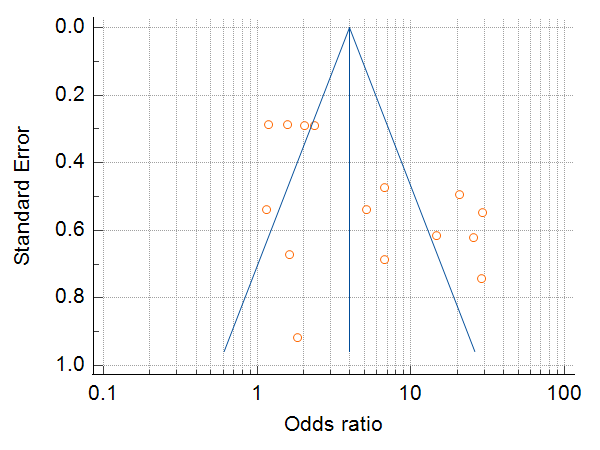

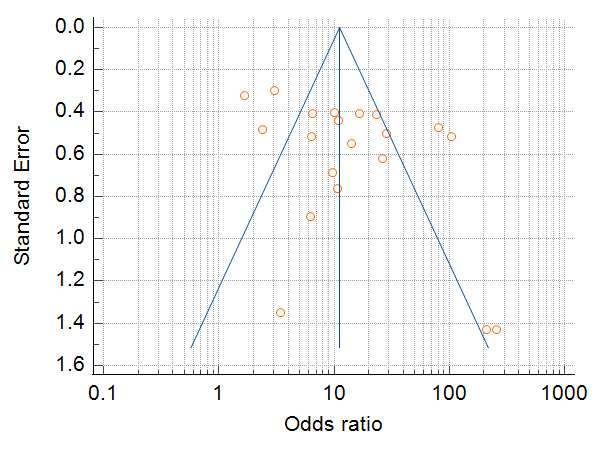


**A**

**B**

**Figure S1:** Funnel plots of Guggulsterone versus control: a. at t=24h; b. at t˃24h

**Table S1:** Summary of the *in vivo* studies retrieved, based on the effect of Guggulsterone in various cancer types

| **S. No.** | **Author Name** | **Study Design** | **Animal model characteristics** | | | | **Cancer model** | | | | **Intervention Characteristics** | | | | |
| --- | --- | --- | --- | --- | --- | --- | --- | --- | --- | --- | --- | --- | --- | --- | --- |
|  |  | **Type of control group** | **Species/ strain** | **Age/ weight** | **Immune status** | **Sex** | **Type of Cancer** | **Number of tumor cells** | **Location of injection of tumor cells** | **Model** | **Intervention** | **Route of administration** | **Dose/ Frequency** | **Duration of treatment** | **Pathway**  **of drug action** |
| 1. | Sarfaraz et al., 2008 | - | SENCAR mice | 5–6 weeks old | Immuno-competent | F | Skin  tumorigenesis | - | Dorsal side of the skin | DMBA (50 nmol DMBA in 200μl acetone)- and TPA (3.2 nmol in 200μl acetone)-induced | Guggulsterone | Topical | 0.8, 1.6μM | 20 weeks | Modulation of MAPK and NF-κB pathways |
| 2. | Leeman-Neill et al., 2009 | Saline | Nude mice | 6–8 weeks | Immunocompromised | F | Head and neck cancer | 1 × 10^6^  1483 and UM-22b | left and right flank | Cells injected into animal’s left and right flank, respectively | Guggulsterone/ saline | Oral | 2mg | 6 weeks | Decrease in STAT3 expression |
| 3. | Guan et al., 2014 | - | Nude mice | 6 weeks | Immunocompromised | - | Esophageal cancer | 2 × 10^6^ for 2 days then after that 3 × 10^6^ cells | Right flank | SKGT-4 cells | Guggulsterone | Oral | 50mg/kg | 22 days | Suprress the tumor by 59.6% |
| 4. | Guan et al., 2014 | - | nu/nu nude mice | 6 weeks old | Immunocompromised | M/F | Esophageal cancer | 3 × 10^6^ | Right flank | SKGT-4 cells | Amiloride,  Guggulsterone, or a combination of both | Oral | 5 mg/kg  50 mg/kg | 22 days | Inhibition of NHE-1 expression |
| 5. | Ahn et al., 2012 | - | BALB/c Mice | 4 weeks | Immunocompromised | M | Pancreatic cancer | 10^6^ cells/ mouse | Subcutaneous | MiaPaCa-2 cells | Guggulsterone  Gemcitabine  or a combination of both | Intraperitoneal | 10mg/kg  125mg/kg  10mg/kg, 5 times/wk and 125 mg/kg, 2 times/wk | 5 times/wk for 3 weeks  2 times/wk for 3 weeks  3 week | Apoptosis induction by suppressing  Akt and nuclear factor-κB activity and by modulating apoptosis-related  protein expression |
| 6. | Chen et al., 2021 | - | BALB/cAnN.Cg-Foxn1nu/CrlNarl nude mice | 20–25 g | Immunocompromised | - | Bladder cancer | 1 × 10^6^ | Subcutaneous | TSGH8301 cells (with Matrigel 1:1 mixed) | Guggulsterone Solution | Intraperitoneal | 20 and 40 mg/kg | 2 weeks | Induces apoptosis and inhibits lysosomal-dependent migration |
| 7. | An et al., 2009 | - | nu/nu mice | 6 weeks | Immunocompromised | - | Colon cancer | 1×10^7^ | Subcutaneous | HT-29 cells were injected | Gugguls-terone | Intraperitoneal | 20 and 40 mg/kg | 2 weeks | Inhibits the growth of HT-29 induced tumor |
| 8. | Xiao and Singh, 2008 | vehicle (PBS) | nude mice | 5–6 weeks old | Immunocompromised | M | Prostate cancer | 3 × 10^6^ | Subcutaneous | DU145 cells | Gugguls-terone | Oral | 40 mg/kg | 4 weeks | Inhibits angiogenesis |
| 9. | Xu et al., 2014 | DMSO | Athymic nude mice and BALB/c (nu + nu + genotype) | 4–6-weeks-old, weighing 20–25 g | Immunocompromised | - | Breast cancer | 1 × 10^7^ | Subcutaneous | MCF-7 cells | Gugguls-terone  Doxorubicin | Intraperitoneal | 30 mg/kg  5 mg/kg | Twice a week for 2 weeks | Reversal of doxorubicin resistance |

**Table S2:** ToxR reliability assessment scores for individual *in-vitro* studies

| **S. No.** | **Authors** | **Criteria group I (Mean ± SD)** | **Criteria group II (Mean ± SD)** | **Criteria group III (Mean ± SD)** | **Criteria group IV (Mean ± SD)** | **Criteria group V (Mean ± SD)** | **Combined score (Mean ± SD)** | **Weighted score (Mean ± SD)** | **Initial category (Numerical result)** | **Revised categories after checking red scores** |
| --- | --- | --- | --- | --- | --- | --- | --- | --- | --- | --- |
| 1. | Macha et al., 2010 | 3.00 ± 0.00 | 3.00 ± 0.00 | 6.00 ± 0.00 | 3.00 ± 0.00 | 2.00 ± 0.00 | 17.00 ± 0.00 | 5.00 ± 0.00 | 1 | 1 |
| 2. | Gottardi et al., 2006 | 3.00 ± 0.00 | 3.00 ± 0.00 | 6.00 ± 0.00 | 3.00 ± 0.00 | 2.00 ± 0.00 | 17.00 ± 0.00 | 6.00 ± 0.00 | 1 | 1 |
| 3. | Yamada et al., 2010 | 3.00 ± 0.00 | 3.00 ± 0.00 | 6.00 ± 0.00 | 3.00 ± 0.00 | 2.00 ± 0.00 | 17.00 ± 0.00 | 6.00 ± 0.00 | 1 | 1 |
| 4. | Lv et al., 2021 | 2.00 ± 0.00 | 3.00 ± 0.00 | 6.00 ± 0.00 | 3.00 ± 0.00 | 2.00 ± 0.00 | 16.00 ± 0.00 | 6.00 ± 0.00 | 1 | 1 |
| 5. | Ahn et al., 2012 | 3.00 ± 0.00 | 3.00 ± 0.00 | 6.00 ± 0.00 | 3.00 ± 0.00 | 2.00 ± 0.00 | 17.00 ± 0.00 | 6.00 ± 0.00 | 1 | 1 |
| 6. | Zhong et al., 2015 | 3.00 ± 0.00 | 3.00 ± 0.00 | 6.00 ± 0.00 | 3.00 ± 0.00 | 2.00 ± 0.00 | 17.00 ± 0.00 | 6.00 ± 0.00 | 1 | 1 |
| 7. | An et al., 2009 | 3.00 ± 0.00 | 3.00 ± 0.00 | 6.00 ± 0.00 | 3.00 ± 0.00 | 2.00 ± 0.00 | 17.00 ± 0.00 | 6.00 ± 0.00 | 1 | 1 |
| 8. | Rebecca Leeman et al., 2009 | 3.00 ± 0.00 | 3.00 ± 0.00 | 6.00 ± 0.00 | 3.00 ± 0.00 | 2.00 ± 0.00 | 17.00 ± 0.00 | 6.00 ± 0.00 | 1 | 1 |
| 9. | Leo et al., 2019 | 4.00 ± 0.00 | 3.00 ± 0.00 | 6.00 ± 0.00 | 3.00 ± 0.00 | 2.00 ± 0.00 | 18.00 ± 0.00 | 6.00 ± 0.00 | 1 | 1 |
| 10. | Chen et al., 2021 | 4.00 ± 0.00 | 3.00 ± 0.00 | 6.00 ± 0.00 | 3.00 ± 0.00 | 2.00 ± 0.00 | 18.00 ± 0.00 | 6.00 ± 0.00 | 1 | 1 |
| 11. | Dixit et al., 2013 | 3.00 ± 0.00 | 3.00 ± 0.00 | 6.00 ± 0.00 | 3.00 ± 0.00 | 2.00 ± 0.00 | 17.00 ± 0.00 | 6.00 ± 0.00 | 1 | 1 |
| 12. | Choudhuri et al., 2011 | 3.00 ± 0.00 | 3.00 ± 0.00 | 6.00 ± 0.00 | 3.00 ± 0.00 | 2.00 ± 0.00 | 17.00 ± 0.00 | 6.00 ± 0.00 | 1 | 1 |
| 13. | Moon et al., 2011 | 3.00 ± 0.00 | 2.00 ± 0.00 | 6.00 ± 0.00 | 3.00 ± 0.00 | 2.00 ± 0.00 | 16.00 ± 0.00 | 6.00 ± 0.00 | 1 | 1 |
| 14. | Shishodia et al., 2007 | 3.00 ± 0.00 | 2.00 ± 0.00 | 6.00 ± 0.00 | 3.00 ± 0.00 | 2.00 ± 0.00 | 16.00 ± 0.00 | 6.00 ± 0.00 | 1 | 1 |
| 15. | Macha et al., 2013 | 3.00 ± 0.00 | 3.00 ± 0.00 | 6.00 ± 0.00 | 3.00 ± 0.00 | 2.00 ± 0.00 | 17.00 ± 0.00 | 6.00 ± 0.00 | 1 | 1 |
| 16. | Zhong et al., 2015 | 3.00 ± 0.00 | 3.00 ± 0.00 | 6.00 ± 0.00 | 3.00 ± 0.00 | 2.00 ± 0.00 | 17.00 ± 0.00 | 6.00 ± 0.00 | 1 | 1 |
| 17. | Samudio et al., 2015 | 3.00 ± 0.00 | 2.00 ± 0.00 | 6.00 ± 0.00 | 3.00 ± 0.00 | 2.00 ± 0.00 | 16.00 ± 0.00 | 6.00 ± 0.00 | 1 | 1 |
| 18. | Singh et al., 2005 | 3.00 ± 0.00 | 3.00 ± 0.00 | 6.00 ± 0.00 | 3.00 ± 0.00 | 2.00 ± 0.00 | 17.00 ± 0.00 | 6.00 ± 0.00 | 1 | 1 |
| 19. | Tian et al., 2021 | 3.00 ± 0.00 | 3.00 ± 0.00 | 6.00 ± 0.00 | 3.00 ± 0.00 | 2.00 ± 0.00 | 17.00 ± 0.00 | 6.00 ± 0.00 | 1 | 1 |
| 20. | Shi et al., 2015 | 3.00 ± 0.00 | 3.00 ± 0.00 | 6.00 ± 0.00 | 3.00 ± 0.00 | 2.00 ± 0.00 | 17.00 ± 0.00 | 6.00 ± 0.00 | 1 | 1 |
| 21. | Xu et al., 2011 | 3.00 ± 0.00 | 2.00 ± 0.00 | 6.00 ± 0.00 | 3.00 ± 0.00 | 2.00 ± 0.00 | 16.00 ± 0.00 | 6.00 ± 0.00 | 1 | 1 |
| 22. | Xu et al., 2017 | 3.00 ± 0.00 | 3.00 ± 0.00 | 6.00 ± 0.00 | 3.00 ± 0.00 | 2.00 ± 0.00 | 17.00 ± 0.00 | 6.00 ± 0.00 | 1 | 1 |
| 23. | Yamada et al., 2014 | 3.00 ± 0.00 | 3.00 ± 0.00 | 6.00 ± 0.00 | 3.00 ± 0.00 | 2.00 ± 0.00 | 17.00 ± 0.00 | 6.00 ± 0.00 | 1 | 1 |
